# Comparison between MACSprep™ forensic sperm microbead kit and Erase Sperm Isolation kit for the enrichment of sperm fractions recovered from sexual assault samples

**DOI:** 10.1007/s00414-022-02861-7

**Published:** 2022-06-30

**Authors:** Frederic Grosjean, Marylou Favre, Vincent Castella

**Affiliations:** Forensic Genetics Unit, University Center of Legal Medicine, Lausanne – Geneva, Lausanne University Hospital and University of Lausanne, Chemin de la Vulliette 4, 1000 Lausanne 25, Switzerland

**Keywords:** Sexual assault samples, Spermatozoa separation, Forensic genetics, STR profile, Differential lysis, DNA extraction

## Abstract

Sexual assault samples often contain mixtures of cells coming from at least two donors. Ideally, one would need to separate the cells into two cellular fractions: one consisting of the alleged aggressor’s spermatozoa (the sperm fraction) and the other containing the victim’s epithelial cells (the non-sperm fraction). This separation increases the probability of obtaining the alleged offender’s autosomal DNA profile. However, spermatozoa are often collected along with an excess of biological material originating from the victim, and with unfavorable male:female biological material ratios, the absence of separation could result in the PCR amplification of the victim’s DNA profile only. Several approaches are available to enrich/purify the spermatozoa present on sexual assault samples. In this paper, we compare a new method, the MACSprep™ Forensic Sperm MicroBead Kit (MACSprep, based on microbeads conjugated with antibodies bound to spermatozoa and their retention within a magnetic column) with the Erase Sperm Isolation Kit (Erase, a standard differential lysis separation procedure combined with a specific removal of free DNA) routinely used in our lab. The performance of both kits was tested using sets of vaginal and buccal swabs loaded with different dilutions of sperm, or azoospermic semen, representing a total of 120 independent samples. For the samples containing undiluted sperm, an average recovery of 58% was observed for the MACSprep’s sperm fractions and 43% for Erase’s. Significantly better recovery of azoospermic semen was observed in MACSprep’s non-sperm fractions (~ 85%) compared to Erase (~ 28%). Erase performed significantly better than MACSprep in terms of recovery for diluted sperm samples (1:10 to 1:800 sperm dilutions) in the presence of vaginal cells, while the purities of the achieved sperm fractions were in favor of MACSprep for the highest sperm dilutions tested. Similar trends were observed with buccal swabs loaded with 1:200 sperm dilutions. Increased sperm dilutions on vaginal swabs resulted in higher variability in the male material recovered, whatever the separation method used. Both methods were easy to perform and resulted in male DNA extracts ready to use in less than 2 h. Both kits showed their specificities in terms of recovery efficiency and purity of the sperm fractions. Ideally, additional experiments should be performed in different laboratories, using workflow and chemistries different than ours, to better define the peculiarities observed with MACSprep for high dilutions. Improving the recovery of MACSprep for diluted samples, in addition to its better purity observed in the experiments performed, could make it a method of choice for laboratory workflow, despite MACSprep’s current price per sample being about twice the price of Erase’s.

**Supplementary Information:**

The online version contains supplementary material available at 10.1007/s00414-022-02861-7.

## Introduction

Sexual assault samples are particularly challenging to analyze because they often contain spermatozoa among a larger proportion of cells originating from the victim (who could be either a woman or a man) than from the offender, in a generally unfavorable ratio for a direct spermatozoa-derived STR amplification. The gender of the victim has no importance as long as the only sperm found on the samples originates from the alleged offender. When large quantities of the victim’s cells (epithelial cells, macrophages, lymphocytes, neutrophils…) are present on such samples, a direct lysis most often results in the preferential autosomal amplification of the victim’s DNA (the major female or male contributor) and thus masks the alleged offender’s DNA profile. This typically occurs when the minor contributor represents less than 5% to 10% of the total amount of DNA extracted from such a sample. This limit has been reported in other studies [1–4] and is also the lower detection limit observed in our laboratory (internal validation studies), when using NGM SElect amplification.

In absence of biological material separation, when the alleged aggressor is a male and the victim is a female, the analysis of Y chromosome-specific STRs (Y-STRs) may give access to a part of the male contributor’s genetic information [5–8]. Y-STRs are male-specific STRs and therefore their amplification should not be affected by the presence of female DNA [9, 10], although some alleles have been reported to be more difficult to call with extremely unbalanced mixture samples, such as 1:32,000 (male:female) (Promega, PowerPlex Y23 product sheet). However, the discrimination of individual with Y-STRs is much less efficient than with autosomal STRs, as all the males from a given paternal lineage share the same inherited Y-STR haplotype (unless a mutation has occurred). Y-STR profiles can be used locally or can be submitted to Y-STR national databases, wherever available, for comparison with Y-STR profiles of known individuals, or possibly to provide links between cases. Rapidly mutating Y-STRs (RM-YSTR) amplification offers a chance to differentiate related males [11–16], as they amplify another set of Y-STRs known to have a higher mutation rate (10^−2^ and higher).

Another approach to differentiate a minor contributor in unbalanced mixture samples, in the absence of biological material separation, lies in the use of DIP-STR markers [17–20]. DIP (deletion-insertion polymorphism)-STR are sequences found in the human genome where DIP polymorphisms (long or short, depending on the polymorphism present) and STRs are close to one another and can be amplified together using DIP-specific primers. When the minor contributor’s DNA possesses a private DIP allele, the corresponding DIP-STR haplotype can be accessed even in severely unbalanced mixtures (up to 1:1000), and this independently of the gender of the major/minor contributors.

Although quite elegant, both RM-YSTRs and DIP-STRs approaches do require reference material from all the contributors possibly at the source of the DNA being analyzed to allow direct comparisons. Another possible limitation of either Y-STRs, RM-YSTRs, or DIP-STRs-amplifications of rape samples, in the absence of separated spermatozoa fraction, is the impossibility to give information on the biological material at the origin of the observed profile. Indeed, the amplified (Y-, RM-Y-, or DIP-) STR markers observed may come from any types of male cells present in the mixture and not only spermatozoa. In a difficult scenario (i.e., presence of several male fluids (semen, blood, skin cells, and/or saliva)), it could be impossible to determine whether the Y-STR profile observed originates from spermatozoa, or from any other male cells present in the sample. For the cases where the analysis of the sperm fractions results in single source male DNA profiles, it seems reasonable to conclude that these profiles originated indeed from sperm. In other multiple contributors’ admixed samples, attributing an observed profile to a given biological fluid may be impossible to achieve.

To circumvent these issues, a separation of the spermatozoa from other cell types needs to be done prior to cell lysis and DNA amplification. This separation results in two distinct fractions, one containing the spermatozoa originating from the alleged offender (the sperm fraction), and the other, the non-spermatozoa biological material originating mainly from the victim (the non-sperm fraction). Several approaches, for the separation of spermatozoa from other cell types, have been developed over the years to process sexual assault samples (differential lysis [21–24], sieve-based filtration [25, 26], laser micro-dissection [27–32], micro-fluidic devices [33, 34], flow cytometry [35–37], acoustic trapping device [38], capillary zone electrophoresis [39], DEP-array sorting [40–43], antibody-based separation [44–47]). They can result in the autosomal STR profiles of the alleged offenders. These profiles can then be submitted to national, or international, DNA databases and be compared with the DNA profiles of known individuals and/or possibly to provide links between cases.

The processing of samples using the above methods within a forensic framework might be hampered by the following drawbacks:

- The separation devices may only be at a prototype stage and not yet available on the market (i.e., micro-fluidic devices).
- Contamination issues with degraded material and cell-free DNA present in the original sample may impair the quality of the obtained profiles (i.e., sieve-based separation, flow cytometry).
- An automated approach may not be available (e.g., differential lysis, laser micro-dissection).
- The equipment may be expensive, require a dedicated operator, and/or have low throughput (laser micro-dissection, flow cytometry, and DEP-array are such examples).

Erase Sperm Isolation Kit (PTC laboratories, Columbia, MO, USA) is a commercial method, based on differential lysis, routinely used in our laboratory. It relies on a two-step lysis procedure, first lysing all the non-spermatozoa cells, then increasing the lysis strength with the addition of DTT, to break the disulfide bounds found on the spermatozoa’s head and release their DNA (Erase Sperm Isolation Kit manual). A centrifugation step takes place, following the first lysis, to pellet down the spermatozoa and remove the supernatant (containing the non-spermatozoa cells free DNA). The spermatozoa pellet is subsequently treated with a deoxyribonuclease in order to digest the remaining free DNA originating from the lysed non-spermatozoa cells. This procedure normally ensures that only spermatozoa-derived DNA is found in the sperm fraction extract after the second lysis. Despite the deoxyribonuclease step prior to spermatozoa’s lysis, some challenging situations may occur when the amount of the victim’s cells are much more abundant than the alleged aggressor’s spermatozoa, leaving significant quantities of non-lysed free-DNA from the victim’s cells in the supernatant, on top of the low numbers of pelleted spermatozoa. When such sperm fractions are amplified, the remaining victim’s free DNA still present may mask, or compete with, the amplification of the alleged offender’s DNA, resulting in difficult to interpret DNA profiles or, in some extremely unbalanced samples, resulting in the amplification of the victim’s DNA only.

The MACSprep Forensic Sperm MicroBead Kit (Miltenyi Biotec, Bergisch Gladbach, Germany, Europe) separation technique specifically retains spermatozoa within a magnetic column following the recognition and binding of spermatozoa by specific antibodies coupled to magnetic beads (MACSprep Forensic Sperm MicroBead Kit manual). While retained within the column, the spermatozoa can be washed, thus removing non-spermatozoa cells and possible free DNA originating from the victim’s lysed cells. This should result, once the spermatozoa have been flushed out of the column (as the sperm fraction) and lysed, in DNA profiles originating from spermatozoa only.

The expected purity achievable with the MACSprep’s separated samples, and its apparent ease of use, led us to compare this separation method with our current separation method (Erase). Both separation kits were compared in terms of recovered material and purity of the sperm fractions, specificity, the ease of use, and hands-on time needed per sample, to seek the possible advantages of one of those two methods over the other.

## Material and methods

### Swab preparation

4N6 FLOQSwabs Genetics (Copan, Brescia, Italy) were loaded with 20 µl of undiluted sperm or 20 µl of 1:200 PBS-diluted sperm from a voluntary donor, or 20 µl of azoospermic semen from a voluntary donor. For each condition, six swabs were prepared in parallel. Once the (diluted) sperm, or azoospermic semen, was deposited on the swabs, they were left to dry overnight at room temperature before being returned in their protective case, and kept at room temperature until used.

Two voluntary informed participants took vaginal swabs (4N6 FLOQSwabs Genetics, Copan) over a period of 10 days. Samples were collected with at least 2 h intervals in between two successive collections, with a maximum of 6 samplings per day. A total of 78 samples were collected. After collection, the swabs were left open to dry overnight and then returned to their protective cases and kept at room temperature. Once all the samples were available, a fresh sperm sample was obtained from a voluntary participant, and a sample of frozen azoospermic semen from a vasectomized volunteer was thawed before being used. Serial dilutions of the sperm sample with PBS were made (1:10, 1:100, 1:200. 1:400, 1:800, and 1:1600) before 20 µl of each dilutions were deposited on two sets of three randomly chosen vaginal swabs. Twenty microliters of undiluted sperm was also deposited on two sets of three randomly chosen vaginal swabs. Twenty microliters of azoospermic semen was directly deposited, without dilution, on two other sets of three randomly chosen vaginal swabs. Once the sperm, or azoospermic semen, was added, the swabs were left to dry overnight, before being returned to their protective cases, and stored at − 20 °C, in order to follow the analytical flow of real sexual assault samples within our laboratory (the samples are kept frozen until they are analyzed, in order to prevent DNA degradation). All samples were then analyzed within 14 days. In order to evaluate the recovery efficiency, 20 µl aliquots of each of the different sperm dilutions used, as well as 20 µl aliquots of azoospermic semen, were kept in Eppendorf tubes, extracted, quantified, as described below, and further compared with the quantified male material recovered from each of the vaginal swabs.

Twelve mock samples were also voluntarily prepared using buccal swabs, provided by a single informed female donor (different from the vaginal swab donors) by rubbing the inside of each cheek with 4N6 FLOQSwabs Genetics for at least 10 s on each side. Those swabs were subsequently loaded with 20 µl of a 1:200 PBS-diluted solution of sperm in triplicates for both MACSprep and Erase separation kits. Once loaded with sperm, the swabs were left to dry at room temperature overnight before being returned in their protective cases and stored at − 20 °C until they were analyzed.

### Spermatozoa separation

For each experiment, the spermatozoa separations were carried out while following the procedures described in the respective Erase and MACSprep provided manuals.

For both separation methods, the cell-containing swabs’ heads were initially cut in 4–5 pieces before being transferred into spin baskets for the first lysis/recovery steps. This was done to increase the mechanical shear forces during the thermomixer initial incubation step, and resulted in increased material being released from the swabs (internal studies, data not shown).

For Erase processed samples, 400 µl of extraction buffer and 7 µl of the provided proteinase K were added to the swab’s head pieces in a 1.5 ml Eppendorf tube, vortexed for 20 s, and incubated for 60 min at 56 °C on an Eppendorf comfort thermomixer at 550 rpm. A rapid spin down was performed before the content of the Eppendorf tube was transferred into a DNA IQ spin basket (Promega, Madison, WI, USA) on a new Eppendorf tube and centrifuged for 5 min at 20,000 g on a 5417R Eppendorf centrifuge. A total of 350 µl of the supernatant was then transferred to a new Eppendorf tube and kept as the “non-sperm fraction﻿.” Ten microliters of the provided solutions 1 and 2 were added to the 50 µl remaining on top of the spermatozoa pellet before it was resuspended by pipetting up and down a few times, and left to incubate under agitation (550 rpm) for 15 min at 37 °C on an Eppendorf thermomixer. Ten microliters of the provided solution 3 was eventually added to the cell suspension, before it was briefly vortexed and incubated for an additional 15 min under agitation (550 rpm) at 56 °C on an Eppendorf thermomixer. This resulted in the lysis of the spermatozoa and was kept as the “sperm fraction﻿.”

For MACSprep, 600 µl of freshly prepared solution 1 was added onto the swab head pieces in a 1.5 ml Eppendorf tube (A), vortexed for 10 s, and incubated for 30 min at 25 °C on an Eppendorf comfort thermomixer at 800 rpm. The sample was then vortexed for 10 s and a rapid spin down was performed before the swab head pieces and the solution 1 were transferred into a DNA IQ spin basket (Promega) onto a new 1.5 ml Eppendorf tube (B). One hundred microliters of solution 1 was used to rinse the first Eppendorf tube (A) and was transferred into the spin basket containing the swab head pieces. The Eppendorf tube (B) was then centrifuged, along with the spin basket, for 5 min at 16,000 g. The spin basket containing the swab head pieces was further discarded. The supernatant was kept in a new Eppendorf tube as the “non-sperm fraction.” The sperm pellet was suspended in 60 µl of freshly prepared solution 2, well mixed with 40 µl of anti-spermatozoa microbead-coupled antibodies and incubated for 15 min at 25 °C without agitation. MS columns were prepared by running 2 × 500 µl of MACSprep Forensic Buffer through them, while standing on a MACS Separator Magnet, before new Eppendorf tubes were placed under the columns and the cell suspensions containing the antibody-bound spermatozoa were loaded into them. Particular care was taken when loading the stained spermatozoa containing cell solution, in order to deposit it directly on top of the visible magnetic beads in the column, and avoid spattering on the column wall. Each of the Eppendorf tubes used for the spermatozoa’s staining (B) was washed once with 400 µl of MACSprep Forensic Buffer, which was then also transferred into the corresponding MS column. Unlabeled cells present in the cell suspension were collected in the flow-through. MS columns were washed twice with 500 µl of MACSprep Forensic Buffer before they were removed from the MACS Separator and placed onto new Eppendorf tubes (C). Five hundred microliters of MACSprep Forensic Buffer was then added into the column and the content was flushed out of the column by pushing down the provided plunger. This resulted in the “sperm fraction.”

### Samples extraction and purification

DNA from the different sperm and non-sperm fractions, collected with either Erase or MACSprep kits, were extracted and purified using the QIAamp DNA Mini Kit (Qiagen) in the presence of DTT and 20 µl of the provided proteinase K. Briefly, 200 µl of AL buffer and 10 µl of DTT solution (7.7 mg DTT (AppliChem, DTT BioChemica, A1101) in 50 µl H2O) were added to 200 µl of the different fractions (20 µl of the provided proteinase K was also added to the MACSprep fractions) before being incubated for 45 min at 56 °C on an Eppendorf comfort thermomixer at 450 rpm. This was followed by a rapid spin down and the addition of 200 µl of 96% EtOH to each sample. The solution was then transferred onto a QIASpin column placed onto a collection tube. A first centrifugation at 6800 g for 1 min was performed before the collection tube was exchanged for a new one and 500 µl of the provided AW1 solution was added into the column. Another 1 min centrifugation at 6800 g was done before the collection tube was changed again and 500 µl of the provided AW2 solution was added into the column. A third centrifugation at 20,000 g was done for 3 min before the collection tube was emptied and a new 20,000 g centrifugation was done for an additional 1 min. Finally, the column was placed onto a new 1.5 ml Eppendorf tube and 60 µl of the provided AE elution buffer was added, incubated for 1 min at RT °C, and centrifuged for 1 min at 6800 g.

### Samples quantification

Two microliters of each extracted DNA from the different fractions was quantified with the Quantifiler™ Trio kit (Thermo Fisher, Applied Biosystems, Waltham, MA, USA), using an ABI 7500 Real-Time PCR system and the HID Real-Time analysis software V1.3 following standard procedures but in half reaction volumes.

The quantification of one negative and two positive controls were done with each run of quantification to ensure that it performed as expected and that no problem took place during the process.

### STR amplification of sperm fractions

Sperm fractions were further amplified using the NGM SElect kit (Applied Biosystems), using a DNA extract target between 0.5 and 1 ng, or 10 µl of the DNA extract if its concentration was 0.05 ng/µl or less. The amplification of one negative and one positive control was done along to ensure that no problems occurred during the process.

DNA profiles were obtained by running 1 µl of the amplification products, mixed with 9.5 µl of formamide and 0.5 µl of GS 600 LIZ, on an ABI3500xL Genetic Analyzer (Applied Biosystems). Data were further analyzed using the GeneMapperID-X V1.5 software (Applied Biosystems). Only alleles with a signal intensity above 75 rfu were considered, with a global cut-off value of 5% for all the allele size ranges.

### Statistical analysis

The mean amounts of male DNA quantities, or the mean male DNA recoveries, obtained during the different experimental setups with the two separation methods (MACSprep and Erase) were compared using pairwise *t*-test, with the analysis ToolPak in Excel 2016. A significance level of *p* < 0.05 was used for all tests.

## Results

### Material recovery

**Fig. 1:**
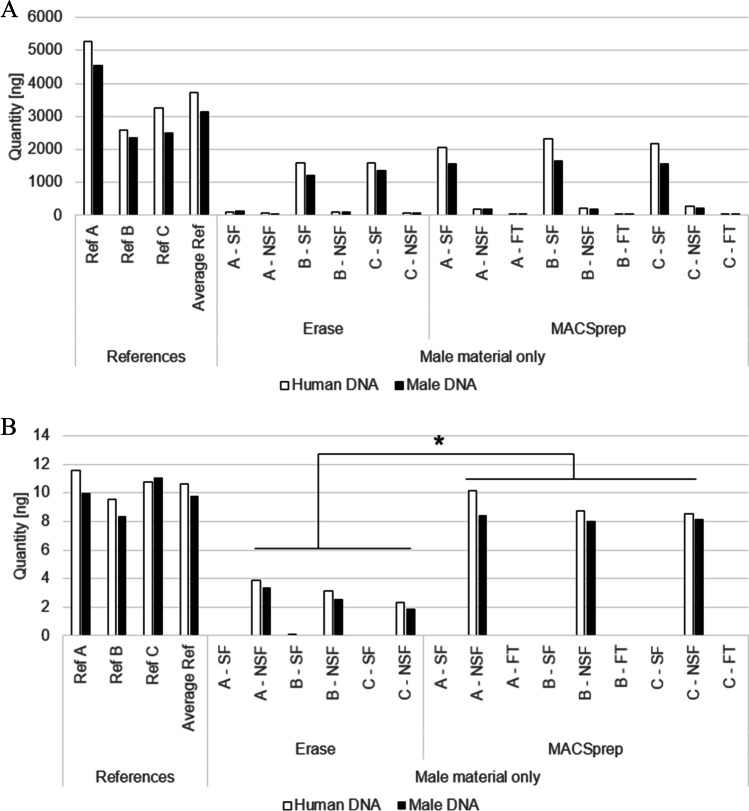
**A** Male material recovered from sperm-loaded swabs using either MACSprep or Erase separation kits. Twenty microliters of undiluted sperm was loaded on swabs and recovered using either one of the separation kits. The material in the different fractions (sperm fraction (SF), non-sperm fraction (NSF), and flow-through (FT)) was quantified and compared to references (20 µl of the same sperm solution in Eppendorf, lysed and quantified). A, B, and C stand for triplicate. (**B**) Male material recovered from azoospermic semen-loaded swabs using either MACSprep or Erase separation kits. Twenty microliters of azoospermic semen was loaded on swabs and recovered using either one of the separation kits. The material in the different fractions (sperm fraction (SF), non-sperm fraction (NSF), and flow-through (FT)) was quantified and compared to references (20 µl of the same azoospermic semen solution in Eppendorf, lysed and quantified). A, B, and C stand for triplicate. Asterisk indicates a significant difference in quantities of recovered material between both methods (*p* < 0.05)

In order to compare both separation kits’ efficiency in regards of spermatozoa separation, swabs were loaded with either 20 µl of undiluted sperm, or 20 µl of undiluted azoospermic semen in the absence of vaginal cells. Triplicates of 20 µl of each seminal liquid used were extracted and quantified as references. Triplicates of each swab, loaded with either undiluted sperm or undiluted azoospermic semen, were then processed with MACSprep and Erase Kits in parallel. The resulting sperm and non-sperm fractions were extracted and quantified. Compared to the references, similar recovery efficiencies were observed for both MACSprep and Erase when processing undiluted sperm samples (Fig. 1A) Fifty to fifty-two percent of the loaded material was found in the MACSprep sperm fractions (SF), and 4–43% in the corresponding Erase sperm fractions. The 4% recovery observed for one of the Erase sperm fraction was an outlier and a laboratory error cannot be excluded.

To ensure the spermatozoa specificity of both separation kits, the same experiment was repeated using 20 µl of azoospermic semen, with no expected material in the sperm fraction this time. Indeed, all material ended up in the non-sperm fractions (NSF) for the two techniques. MACSprep’s NSF resulted in 81–86% recovery of the loaded material, whereas Erase’s NSF contained between 19 and 34% of the loaded material (Fig. 1B). The difference between both kits was significant (*p* < 0.05).

The MACSprep’s flow-through fractions (FT), from the magnetic columns, were kept and their male material were quantified in order to check if part of the difference between the expected material found in the sperm fractions and the reference samples could be explained by material wrongly directed in these fractions during the separation process. As shown in Fig. 1A and B, the flow-through fractions collected during the MACSprep separations (mostly a wash step) were devoid of male material for both undiluted sperm and undiluted azoospermic semen.

### Purity and reproducibility of the sperm fractions

The purer the separated fractions, the more allelic information from the obtained DNA profile can be trusted and the lesser the risk to wrongly assign a given allele to one contributor. To compare how both kits performed regarding purity and reproducibility, eight sets containing each three randomly chosen vaginal swabs were loaded with 20 µl of 1:10 PBS-diluted sperm and another eight sets of three randomly chosen vaginal swabs were loaded with 20 µl of 1:200 PBS-diluted sperm. Four technicians processed one set of each sperm dilutions with Erase and one set of each sperm dilutions with MACSprep. The total DNA and male-only DNA were quantified for each of the sperm fractions obtained and the male DNA/total DNA ratio was calculated for each sperm fraction. The closer this ratio is to 1, the purer the fractions are (i.e., contain male-only biological material).

**Fig. 2:**
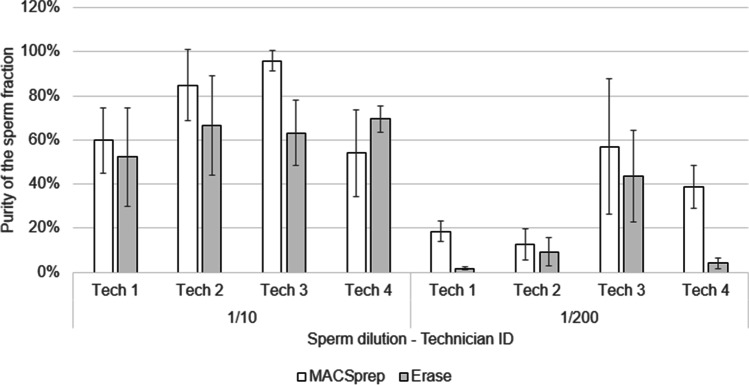
Purity of the different sperm fractions. Vaginal swabs, loaded with either 20 µl of 1:10 PBS-diluted sperm or 20 µl of 1:200 PBS-diluted sperm, were processed with both separation kits in triplicates. Human and male materials were quantified for each sample. The purity of the sperm fraction was calculated as the male DNA contribution to the total human DNA quantified. The closer to 100%, the purer the fraction is, regarding male material. Error bars are from the triplicate processed for each kit by each technician

As shown in Fig. 2, MACSprep resulted generally in higher, or equivalent, levels of purity of the resulting sperm fractions compared to those observed with Erase. In general, 1:10 diluted sperm samples showed purer sperm fractions than 1:200 diluted sperm samples for both kits. MACSprep showed purer sperm fractions than Erase for half of the 1:200 diluted sperm samples. Variability was observed between technicians for both kits. 1:10 PBS-diluted samples processed with Erase produced more reproducible results, with a range of purity between 52 and 69% achieved by the different technicians, but this apparent reproducibility disappeared with the 1:200 PBS-diluted samples. MACSprep ranged from 54 to 96% purity of the sperm fractions for the 1:10 diluted samples and was in general higher than what was observed for Erase. A lot of variability was also observed for the MACSprep 1:200 diluted samples, but with a purity slightly higher than what was obtained for the corresponding Erase samples.

As expected, to obtain male-only material in the sperm fractions became more difficult with higher sperm dilutions loaded on the vaginal swabs. While the two methods showed similar purity of the sperm fractions for the swabs loaded with 20 µl of 1:200 diluted sperm for two of the technicians (Fig. 2, technicians #2 and #3, and Supplementary Table S1), a 10 times higher purity was observed with MACSprep for the other two (Fig. 2, technicians #1 and #4, and Supplementary Table S1) for the same sperm dilution. The increased variability observed for the samples with low amounts of spermatozoa shows a part of the difficulties to master the separation techniques, even for trained technicians, for such samples.

### Influence of biological mixtures on recovery

The ability of both separation kits to successfully separate spermatozoa was subsequently tested using swabs loaded with different types of biological mixtures. Vaginal swabs, buccal swabs, and new, unused swabs were all loaded with 20 µl of 1:200 PBS-diluted sperm. This dilution was chosen as it produced a lot of variability in the previous experiments (Fig. 2). Triplicates of each biological mixture were processed using both kits.

**Fig. 3:**
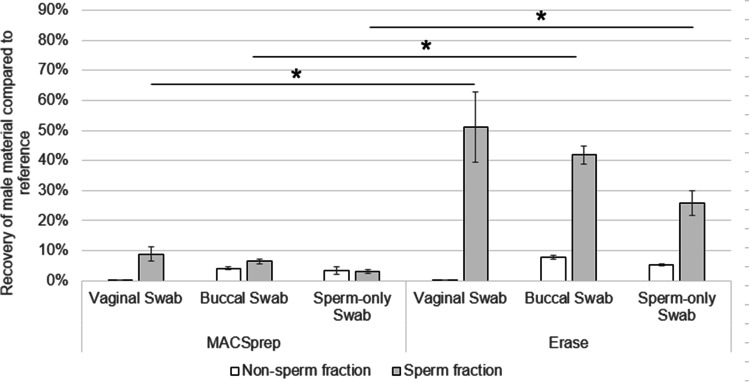
Influence of biological mixture on male material recovery. Vaginal swabs, buccal swabs, and new, unused swabs were loaded with 20 µl of 1:200 PBS-diluted sperm. Triplicates of each conditions were separated using both MACSprep and Erase in parallel. The recovered material was then compared to references (20 µl of the same sperm dilution in an Eppendorf, directly lysed and quantified). Based on the results shown in Fig. 1A, MACSprep flow-through was not quantified. Asterisks indicate significant difference in male material recoveries in the sperm fractions (*p* < 0.05)

Erase resulted in significantly (*p* < 0.05) higher recovery rates (50%, 40%, and 25% respectively) compared to MACSprep (less than 10% for all sets of triplicate) for both vaginal and buccal swabs, as well as for the new swabs loaded with diluted sperm only, compared to references (Fig. 3). Swabs loaded with PBS-diluted sperm only released more material with Erase than with MACSprep, which was unexpected, considering the male recovery similarity for both kits observed for swabs loaded with undiluted sperm only (Fig. 1A). Surprisingly, an efficient male material recovery was more difficult to achieve with buccal swabs compared to vaginal swabs. Both MACSprep and Erase resulted in a decrease of male material recovery for buccal swabs compared to vaginal swabs, while the same amount of diluted sperm was loaded on both types of swabs. The male DNA found within the Erase’s and MACSprep’s buccal swabs’ non-sperm fractions may originate from male epithelial cells, leukocytes, and immature sperm cells, or spermatozoa with a compromised membrane present in sperm. The exact reason why similar male biological material seems to be absent from the vaginal swabs’ non-sperm fractions is unclear and would require yet unknown experimental designs to try to answer it.

**Fig. 4:**
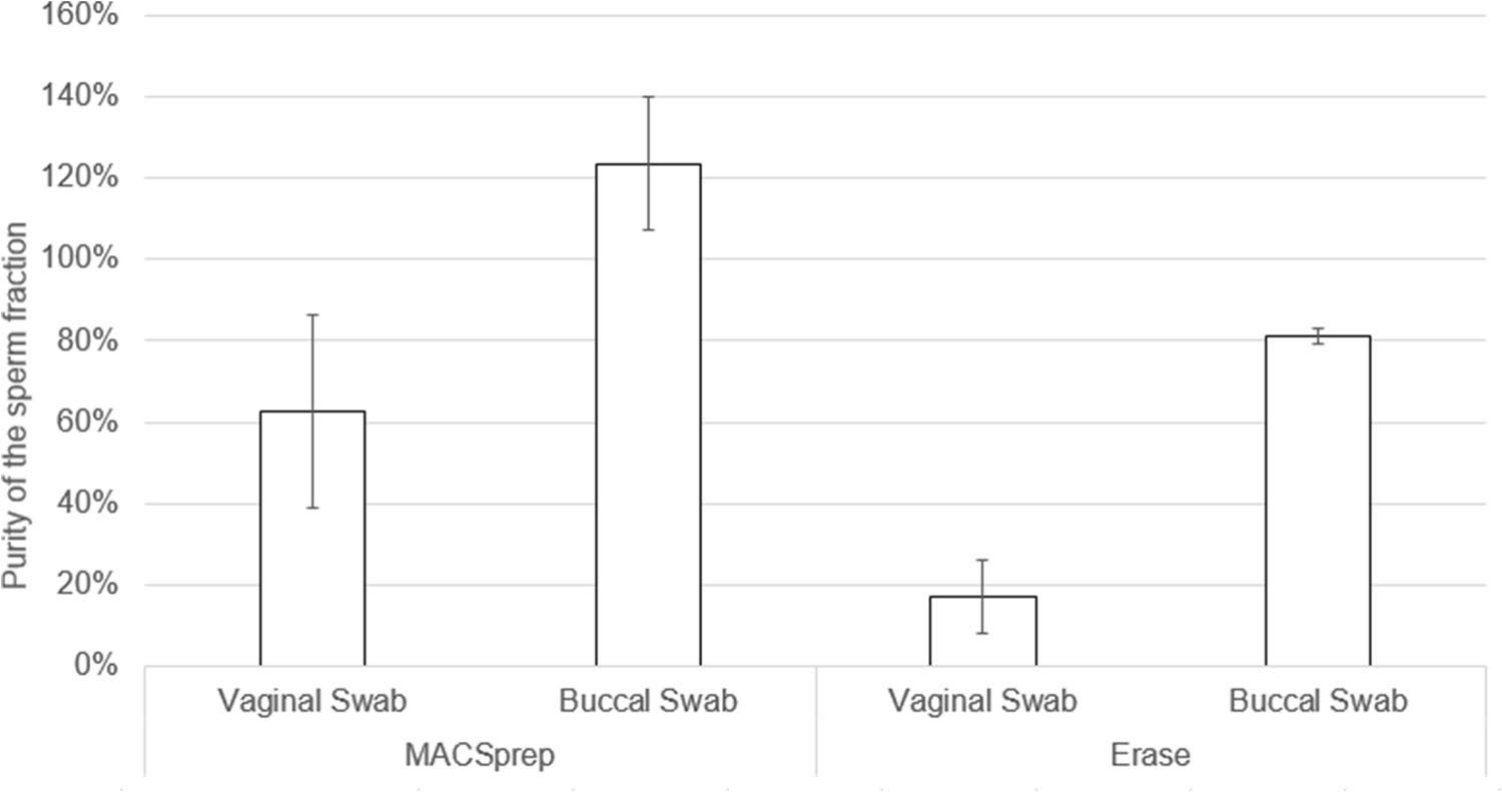
Purity of the sperm fractions in the presence of vaginal or buccal cells. The purity of each of the sperm fraction was calculated for each of the vaginal or buccal swabs loaded with 20 µl of 1:200 PBS-diluted sperm. Error bars are from triplicate processed for each kit

Despite a lower recovery of input male material in the sperm fractions obtained with MACSprep, the purity achieved was higher for MACSprep than what was observed with Erase, independently of the cell types (vaginal or buccal) present on the swabs loaded with 1:200 PBS-diluted sperm (Fig. 4). Both kits resulted in purer sperm fractions when the original cell mixture consisted of buccal cells mixed with diluted sperm, rather than vaginal cells mixed with similar amount of diluted sperm.

### Sensitivity of the kits

**Fig. 5:**
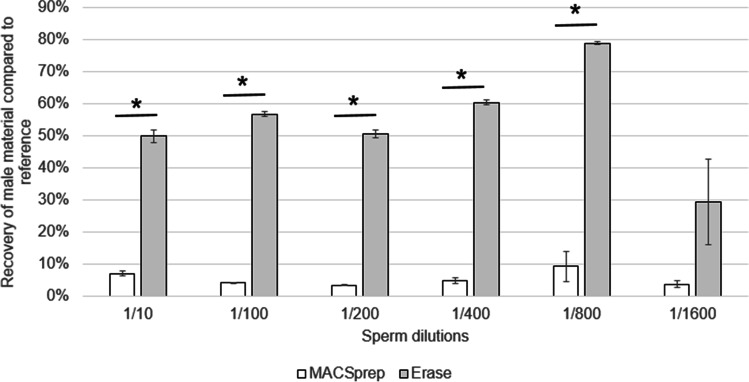
Sensitivity of the kits. Both separation kits were tested on vaginal samples loaded with 20 µl of increasing dilutions of sperm (1:10, 1:100, 1:200, 1:400. 1:800, and 1:1600) in duplicates. The male material in the sperm fractions was quantified and compared to references. Asterisks indicate significant differences in male materiel recovery (*p* < 0.05)

Both kits were further compared with vaginal swabs loaded with 20 µl of sperm with increasing PBS dilutions (1:10, 1:100, 1:200, 1:400, 1:800, and 1:1600) in duplicates. The male material in the sperm fractions was quantified and compared to the expected amount from the corresponding references. Erase performed significantly better (*p* < 0.05), in terms of recovery, than MACSprep for all the dilutions tested, except the 1:1600 dilution (Fig. 5). On average, the male material recovery rate, compared to the loaded reference materials, ranged between 50 and 80% for Erase (except a drop to 30% for the highest dilution tested (1:1600)) and from 5 to 10% for MACSprep.

**Fig. 6:**
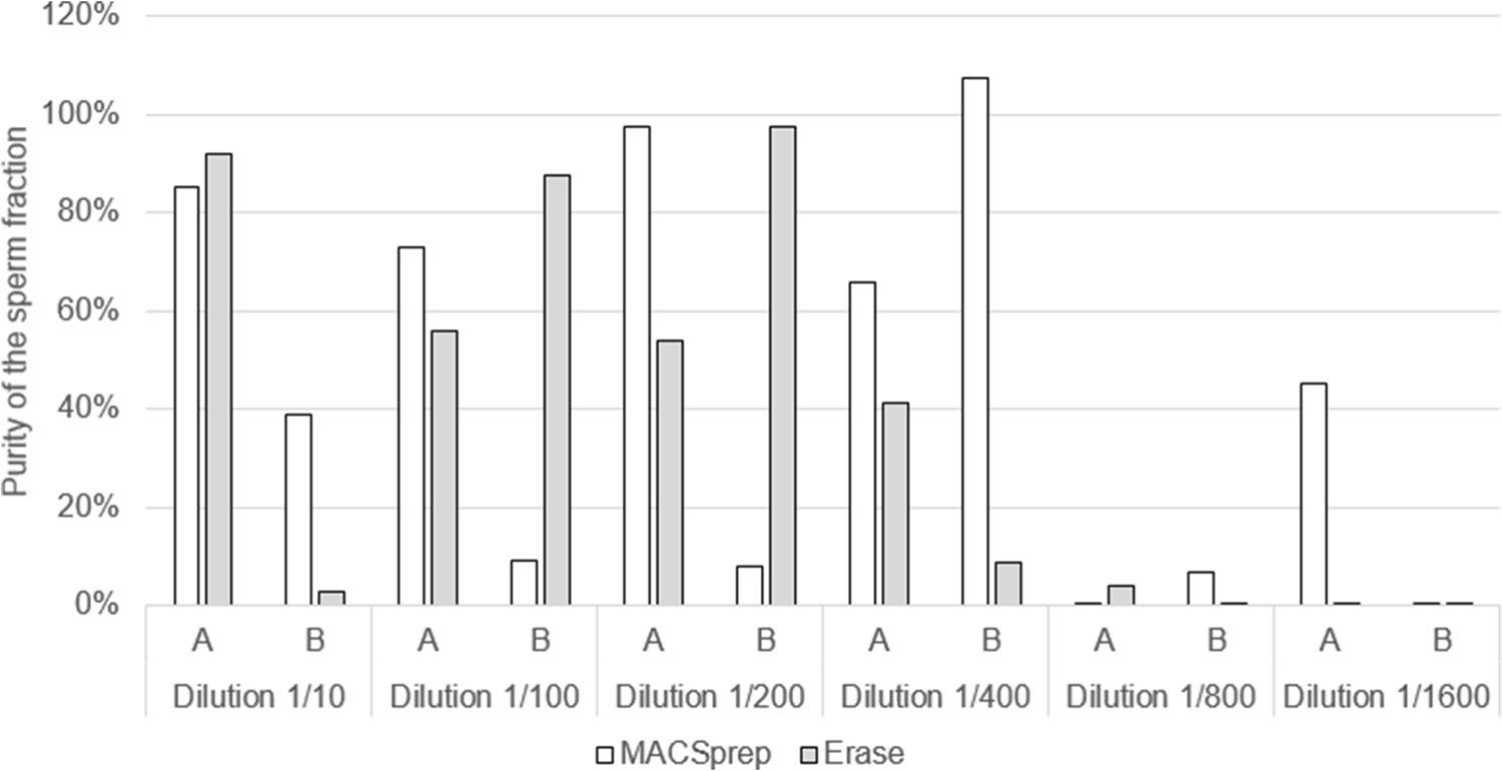
Purity of the sperm fractions with increasing sperm dilutions. Vaginal swabs loaded with 20 µl of the indicated sperm dilutions were separated using both kits in duplicates (A and B). The resulting sperm fractions were extracted and quantified. The purity of the sperm fractions is shown for each sample

The material found in all sperm fractions was quantified for both male and total DNA. A lot of variability was observed between similar sperm dilutions processed with the two different kits. The purity of the resulting male fractions varied from less than 10% to almost 100% throughout the different fractions with no clear trends towards one kit or the other. MACSprep did perform better for the 1:400 and 1:1600 dilutions, compared to Erase. Both kits behaved poorly for the 1:800 dilution (Fig. 6). As expected from the purity observed for the 1:400 sperm dilutions (Fig. 6), NGM SElect amplification resulted in full single male profile from the MACSprep’s sperm fractions. A partial single male contributor was also obtained for one of the sample at 1:1600 sperm dilution with MACSprep, while no quantifiable DNA was detected in the corresponding Erase samples (Supplementary Table S2).

## Discussion

In this study, we tested the new MACSprep™ Forensic Sperm MicroBead Kit and compared it with the Erase Sperm Isolation Kit, routinely used in our laboratory, for their respective abilities to separate spermatozoa from other cell types present on mock sexual assault samples. Both kits are commercially available, with easy to follow instructions. Hands-on time was similar, whichever kit was used, and amplification-ready DNA extracts were available with both methods in less than 2 h.

DNA recovery is known to be influenced by the swab type as well as the extraction method used [48]. An additional loss of material present on a swab is also known to occur when separation takes place prior to DNA extraction, and such losses of male material have been reported during the analysis of sexual assault samples [1]. This was observed for both kits, where an average of 58% of the undiluted sperm loaded on the swabs was recovered in the absence of vaginal cells with MACSprep and an average of 43% with Erase (Fig. 1A). Material was lost during the separation process for both kits and the quantification of the different supernatants for both separation processes did not point to where this loss of material may have occurred. It may originate from cells that did not detach from the swabs, from free DNA adhering to one or more substrate(s) during the separation process, or from yet other unknown phenomena. This would require the design of specifically dedicated experiments to answer this particular question.

Interestingly, when the swabs were loaded with undiluted azoospermic semen, 85% of the loaded material was recovered on average with MACSprep, while only 28% on average were recovered with Erase (Fig. 1B). The difference between sperm and azoospermic semen being primarily the presence of spermatozoa in the ejaculate, it may be possible that the spermatozoa do adhere more to the swabs than the other cell types present in azoospermic semen. This could result in the lower recovery observed for the sperm-loaded MACSprep samples (~ 58%) compared to the azoospermic semen samples (~ 85%). Staining the swabs, after the initial recovery step, with DNA-specific probes (i.e., DAPI or PI) or using picroindigocarmine along with nuclear fast red, and screening for the presence of cells and/or nucleus still attached to the swabs with a microscope (equipped with UV source if DAPI/PI are used) may be a possibility to challenge this hypothesis. For Erase, the difference of recovery observed between the sperm and azoospermic semen (43% and 28% respectively) may come from a yet unknown different origin, as the absence of spermatozoa did not result in an increased recovery of male material. The lysis strength of the kits’ respective buffers used at the beginning of the recovery procedure may also result in a more efficient lysis of the cells other than spermatozoa during the first spermatozoa’ separation step, explaining why azoospermic semen behave differently than sperm with the two kits.

As stated above, the recovery efficiencies were in similar ranges for both kits in the presence of undiluted sperm, but were much higher for Erase (from 29 to 78%) with all PBS-diluted sperm samples compared to MACSprep (from 3 to 9%, Fig. 5). The sudden decrease in recovery observed with MACSprep, once sperm was diluted, may in part be linked to the dilution process itself, affecting somehow the recognition of the spermatozoa by the spermatozoa-specific antibody. This would need to be tested in future experimental designs in order to better understand it and try to increase the corresponding recovery rates of these samples.

Despite lower recovery (Fig. 5), the purity of the sperm fractions was similar between Erase and MACSprep for vaginal swabs loaded with 1:10 sperm dilution (Fig. 2). This purity was higher (up to 10 times), although variable, for MACSprep compared to Erase for half of the vaginal swabs loaded with 1:200 sperm dilution (Fig. 2 and Fig. 4) as well as for buccal swabs loaded with 1:200 diluted sperm (Fig. 4). The purer male-only sperm fractions, obtained with MACSprep, were most likely linked to the column wash step done to remove possible non-spermatozoa material and cell-free DNA from the spermatozoa. Both techniques resulted in a lot of variability for the purity of the sperm fractions when processing samples loaded with lower numbers of spermatozoa (using higher sperm dilutions, Fig. 6). This may in part be explained by the fact that the number of female cells on those swabs, as well as the amounts of vaginal secretions, was not controlled, from one swab to the other, and may have had an unexpected effect on the efficiency of the male material recovery with both techniques. Preparing similar and homogeneous mock vaginal samples is a challenge in itself which was out of the scope of this study.

Buccal swabs were used, along vaginal swabs, to test the influence of the cell mixtures on the spermatozoa’ separation with the two kits. Despite the fact that buccal swabs may not be part of typical sexual assault swabs collected, it was also interesting to challenge them with both MACSprep and Erase as, in some particular cases, samples following a forced fellatio may be part of the sexual assault kits transferred for analyses. Purer sperm fractions were obtained, post-separation with both kits, for buccal swabs loaded with PBS-diluted sperm, compared to vaginal swabs loaded with the same amount of the same PBS-diluted sperm. This may in part be explained by the sample preparation. Sperm dilutions were loaded on buccal swabs right after they were collected while they had no yet had time to dry. For vaginal swabs, on the other hand, as the samples were collected over a long period of time, they were stored dry until they were all available and subsequently loaded with the same sperm dilutions at the same time. The difference between these two types of samples may have resulted in a multi-layer kind of cell deposition for the vaginal swabs, compared to a more homogeneous cell mixture for the buccal swabs. The remaining absorption capacities associated with these two types of swabs may have been different when sperm dilutions were deposited onto them. Spermatozoa loaded onto a dried layer of vaginal cells may have been easier to recover than those more homogeneously mixed with buccal cells. Also, one cannot rule out a possible role of the cellular composition and the surrounding non-cell compounds found in those two types of samples, which may interact differently with spermatozoa (approximately 10 times more female material was present on the buccal swabs compared to the vaginal ones used in this study).

Separation of spermatozoa with antibody-based spermatozoa is an active field of research as was shown by different publications using several different antibodies (anti-PH-20 [44], SP-10, NUH-2, HS-21 [45], MOSPD3 [46]) on fresh mixtures of epithelial cells and spermatozoa. Magnetic separation was also recently proposed as a way to isolate spermatozoa from heterogeneous samples using SLeX-conjugated magnetic beads [47] using cultured vaginal cells, or buccal cells, mixed with diluted sperm, but showed carryover of non-spermatozoa cells within the corresponding sperm positive fractions. None of these approaches is currently commercially available and is either not tested on dried swabs [44], or showed significant decrease in efficiency when samples were vortexed [45] or when swabs were kept for more than 10 days before being processed [46]. Erase, routinely used in our laboratory, and MACSprep were successful in separating spermatozoa from dried swabs (as those used in the present paper). Routine flow in the laboratory for sexual assault with Erase involves vortexing steps. Vortexing samples did not show a negative impact on MACSprep samples, as it resulted in recovery efficiencies comparable to those observed with Erase for samples loaded with undiluted sperm. More than a year old samples are routinely analyzed within our laboratory with Erase and result in very good quality profiles. A few several-month-old samples were processed with MACSprep in our laboratory, and in collaboration with other laboratories, and produced full male STR profile in the sperm fractions (these few real case samples were analyzed with the agreement of the Swiss ethical committee, as long as no profiles were disclosed and the samples remained anonymous).

MACSprep and Erase, both being commercially available products, can hardly compete with home-made differential lysis solutions regarding the cost per sample. The price per separation is about twice for MACSprep compared to Erase (Supplementary Table S3).

With both kits’ actual formats, Erase is more interesting than MACSprep in regard to recovery efficiency for samples with low amounts of spermatozoa in the presence of large numbers of epithelial cells. This is different if the purity of the sperm fractions is considered. If the current recovery achieved for highly diluted sperm samples could be increased with MACSprep, the higher purity obtained and its already available automation system would make it a method of choice, or at least worth considering, to process large numbers of sexual assault samples and/or reduce backlogs.

Further tests are planned in order to better understand some of the results observed within our laboratory. An additional inter-laboratory study would help to determine whether some of the unexpected results observed within this study are specific to our laboratory workflow or to the kits used.

## Supplementary Information

Below is the link to the electronic supplementary material.

Supplementary file1 (DOCX 20 KB)

## Declarations

All samples used in this study were provided by informed donors, who voluntarily participated in the experimental protocol described and agreed to the publication of the results. The approval from the Swiss ethical committee was not required for the abovementioned study as it represents a validation study rather than a research project, and as such does not require ethical approval regarding local laws. Moreover, no individual information regarding health, genetic information, or donor-specific data was considered during this experimental setup. No further analyses, than those cited in the present paper, were performed using the DNA extracts obtained from the different experiments described. The presented results cannot identify the different donors.
