# Supplementary material for: Comparison between MACSprep™ forensic sperm microbead kit and Erase Sperm Isolation kit for the enrichment of sperm fractions recovered from sexual assault samples
Source: Int J Legal Med. 2022 Jun 30;137(1):267–78. doi: 10.1007/s00414-022-02861-7 (PMC9816209; doi:10.1007/s00414-022-02861-7)
Supplement: Supplementary file 1 — Supplementary file1 (DOCX 20 KB) [file 414_2022_2861_MOESM1_ESM.docx]

|  | Average purity of the sperm fraction [%] | |
| --- | --- | --- |
| **1:10 sperm dilution** | MACSprep | Erase |
| Technician 1 | 60 | 52 |
| Technician 2 | 85 | 67 |
| Technician 3 | 96 | 63 |
| Technician 4 | 54 | 69 |

|  | Average purity of the sperm fraction [%] | |
| --- | --- | --- |
| **1:200 sperm dilution** | MACSprep | Erase |
| Technician 1 | 19 | 2 |
| Technician 2 | 13 | 9 |
| Technician 3 | 57 | 44 |
| Technician 4 | 39 | 4 |

**Table 1.** **Purity of the male material found in the sperm fractions with MACSprep and Erase for two sperm dilutions (1:10 and 1:200).** A set of triplicate of each sperm dilution was processed using MACSprep, and another set of triplicates using Erase by four different technicians. Purity was generally higher for MACSprep compared to Erase. Technician 1 and 4 showed a 10 times better purity for MACSprep compared to Erase for the 1:200 sperm dilution.

| **Kit** | **Sperm dilution / Replicate ID** | **Profile characterization** | **major contributor # of loci (NGMSElect)** |
| --- | --- | --- | --- |
| Erase | 1 :10 A | Single male contributor | 16/16 |
|  | 1 :10 B | Single female contributor | 16/16 |
|  | 1 :100 A | Major male contributor with a few minor alleles from the female contributor | 16/16 |
|  | 1 :100 B | Single male contributor | 16/16 |
|  | 1 :200 A | Major male contributor with a few minor alleles from the female contributor | 16/16 |
|  | 1 :200 B | Single male contributor | 16/16 |
|  | 1 :400 A | Major male contributor with a few minor alleles from the female contributor | 16/16 |
|  | 1 :400 B | Male + female mixture,with a major female contributor | 16/16 |
|  | 1 :800 A | Major female contributor + minor male contributor (full profil) | 16/16 |
| MACSprep | 1 :10 A | Single male contributor | 16/16 |
|  | 1 :10 B | 50:50 male:female mixture | 16/16 |
|  | 1 :100 A | Single male contributor | 16/16 |
|  | 1 :100 B | Major female contributor with almost complete set of alleles from the minor male contributor | 16/16 |
|  | 1:200 A | Major male contributor with a few minor alleles from the female contributor | 16/16 |
|  | 1:200 B | Major female contributor with a few minor alleles from the male contributor | 16/16 |
|  | 1:400 A | Single male contributor | 16/16 |
|  | 1:400 B | Single male contributor | 16/16 |
|  | 1:800 B | Major female contributor with a few minor alleles from the male contributor | 16/16 |
|  | 1:1600 A | Single male contributor | 10/16 |

**Table 2. Characterization of NGMSElect profiles obtained from the different sperm fractions.** The number of exploitable loci, with all alleles called, are indicated for each of the sample analyzed with either Erase or MACSprep. Only the samples with quantifiable DNA amounts were amplified using NGMSElect amplification kit.

|  |  | Price per kit / # tests per kit | Price per test |
| --- | --- | --- | --- |
| Erase | https://www.ptclabs.com/product-category/erase-sperm-isolation-kit/ | $495 / 50 tests | $9.9 |
| MACSprep | https://www.miltenyibiotec.com/US-en/products/macsprep-forensic-sperm-microbead-kit-human.html#130-125-280 | $450 / 25 tests | $18 |

**Table 3. Price per separation using Erase or MACSprep.** Prices shown are those as advertised by each company on their website without any discount. URLs where the information was found for both kits are also indicated.
